# Analysis of V2 Antibody Responses Induced in Vaccinees in the ALVAC/AIDSVAX HIV-1 Vaccine Efficacy Trial

**DOI:** 10.1371/journal.pone.0053629

**Published:** 2013-01-17

**Authors:** Susan Zolla-Pazner, Allan C. deCamp, Timothy Cardozo, Nicos Karasavvas, Raphael Gottardo, Constance Williams, Daryl E. Morris, Georgia Tomaras, Mangala Rao, Erik Billings, Phillip Berman, Xiaoying Shen, Charla Andrews, Robert J. O'Connell, Viseth Ngauy, Sorachai Nitayaphan, Mark de Souza, Bette Korber, Richard Koup, Robert T. Bailer, John R. Mascola, Abraham Pinter, David Montefiori, Barton F. Haynes, Merlin L. Robb, Supachai Rerks-Ngarm, Nelson L. Michael, Peter B. Gilbert, Jerome H. Kim

**Affiliations:** 1 Research Service, Veterans Affairs Medical Center, New York, New York, United States of America; 2 Departments of Pathology and Biochemistry and Molecular Pharmacology, NYU School of Medicine, New York, New York, United States of America; 3 Statistical Center for HIV/AIDS Research and Prevention (SCHARP), Vaccine and Infectious Disease Division of the Fred Hutchinson Cancer Research Center, Seattle, Washington, United States of America; 4 United States Army Material Command-Armed Forces Research Institute of Medical Sciences, Bangkok, Thailand; 5 Human Vaccine Institute, Duke University School of Medicine, Durham, North Carolina, United States of America; 6 U.S. Military HIV Research Program, Walter Reed Army Institute of Research, Silver Spring, Maryland, United States of America; 7 Department of Biomolecular Engineering, University of California Santa Cruz, Santa Cruz, California, United States of America; 8 Theoretical Biology and Biophysics Group, Los Alamos National Laboratory, Los Alamos, New Mexico, United States of America; 9 Vaccine Research Center, National Institute of Allergy and Infectious Diseases, National Institutes of Health, Bethesda, Maryland, United States of America; 10 Public Health Research Institute, University of Medicine and Dentistry of New Jersey, Newark, New Jersey, United States of America; 11 Department of Disease Control, Ministry of Public Health, Nonthaburi, Thailand; 12 Department of Biostatistics, University of Washington, Seattle, Washington, United States of America; Istituto Superiore di Sanità, Italy

## Abstract

The RV144 clinical trial of a prime/boost immunizing regimen using recombinant canary pox (ALVAC-HIV) and two gp120 proteins (AIDSVAX B and E) was previously shown to have a 31.2% efficacy rate. Plasma specimens from vaccine and placebo recipients were used in an extensive set of assays to identify correlates of HIV-1 infection risk. Of six primary variables that were studied, only one displayed a significant inverse correlation with risk of infection: the antibody (Ab) response to a fusion protein containing the V1 and V2 regions of gp120 (gp70-V1V2). This finding prompted a thorough examination of the results generated with the complete panel of 13 assays measuring various V2 Abs in the stored plasma used in the initial pilot studies and those used in the subsequent case-control study. The studies revealed that the ALVAC-HIV/AIDSVAX vaccine induced V2-specific Abs that cross-react with multiple HIV-1 subgroups and recognize both conformational and linear epitopes. The conformational epitope was present on gp70-V1V2, while the predominant linear V2 epitope mapped to residues 165–178, immediately N-terminal to the putative α4β7 binding motif in the mid-loop region of V2. Odds ratios (ORs) were calculated to compare the risk of infection with data from 12 V2 assays, and in 11 of these, the ORs were ≤1, reaching statistical significance for two of the variables: Ab responses to gp70-V1V2 and to overlapping V2 linear peptides. It remains to be determined whether anti-V2 Ab responses were directly responsible for the reduced infection rate in RV144 and whether anti-V2 Abs will prove to be important with other candidate HIV vaccines that show efficacy, however, the results support continued dissection of Ab responses to the V2 region which may illuminate mechanisms of protection from HIV-1 infection and may facilitate the development of an effective HIV-1 vaccine.

## Introduction

The RV144 HIV-1 vaccine trial was the first to demonstrate evidence of protection against HIV-1 infection, with an estimated vaccine efficacy of 31.2% [1]. This vaccine consisted of four doses of a recombinant canary pox priming immunogen, ALVAC-HIV (vCP1521), and two doses of AIDSVAX® B/E, recombinant HIV-1 gp120 proteins from HIV-1 subtype B and circulating recombinant form 01_AE (CRF01_AE).

In order to identify correlates of risk of HIV-1 infection in RV144, two sequential sets of analyses of plasma specimens from study participants were conducted [2]. The first was a series of pilot studies in which 32 types of immunologic assays were performed on sets of plasma and peripheral blood mononuclear cells from uninfected participants who had received either the placebo or the vaccine. Results from the pilot studies were used to select assays for the subsequent case-control study of immune correlates of infection risk. Assays for the case-control study were chosen if the results in the pilot studies showed low false positive rates, a broad dynamic range, low background reactivity, and low specimen volume requirements [2]. Seventeen assay types were selected for the case-control study, and these generated results for 158 variables. To preserve maximal statistical power, six were chosen as primary variables in the case-control study and were analyzed by multivariate analysis. To expand the search for immune correlates, all 158 variables were subsequently evaluated by univariate analyses.

Case-control specimens consisted of specimens drawn two weeks after the last immunization from 41 infected vaccinees (cases) and from 205 frequency-matched uninfected vaccinees (controls). Two of the six primary variables significantly correlated with HIV-1 infection risk in vaccine recipients: 1) The level of plasma IgG antibodies (Abs) reactive with gp70-V1V2, a scaffolded protein carrying the first and second variable regions of an HIV-1 gp120 envelope glycoprotein fused to murine leukemia virus gp70. Levels of Abs specific for gp70-V1V2 were correlated *inversely* with the risk of infection. 2) The level of plasma IgA Abs reactive with a panel of 14 envelope glycoproteins correlated *directly* with risk of infection.

The participation of the V2 region of gp120 in the infectious process and the role of V2-specific Abs in protection from infection have been the subject of investigation and controversy for nearly two decades. Although, by definition, “variable” regions vary in amino acid (AA) sequence, many residues in these regions do not vary, or tolerate only conservative changes [3]. These conserved AAs can form structural elements that result in immunologic cross-reactivity between diverse viruses; for example many Abs specific for variable regions are highly cross-reactive with diverse HIV-1 envelopes [4]–[9]. Moreover, the conserved structural features are required for function. Thus, for example, conserved elements within V2 participate in the formation of the bridging sheet (a constituent of the chemokine receptor binding site [10]–[12]), and V2 contains a tripeptide motif in the mid-loop region of V2 that is a putative α4β7 integrin binding site [13]. Similarly, conserved elements of the third variable region, V3, contribute to its recognition by cross-reactive Abs and to its role in binding to the chemokine receptor [14], [15].

Abs specific for V2 occur in only ∼25–40% of HIV-infected individuals [5], [16]. Interestingly, the cross-reactivity of these Abs does not require extensive mutation from the VH germ line, since V2-specific monoclonal Abs (mAbs) from HIV-infected individuals display a mean mutation frequency of 6.2% from germ line [7] which is comparable to a mean 6.8% mutation frequency found in Abs from normal individuals [17]. Thus, because a large body of data suggests that V2 may be a site of HIV-1 vulnerability, and because a strong Ab response to gp70-V1V2 was correlated with reduced infection in the RV144 clinical vaccine trial, an extensive analysis of all V2 Ab assays used in the RV144 immune correlates study was undertaken and is the subject of this report.

## Methods

### Ethics Statement

The RV144 clinical vaccine trial was registered with ClinicalTrials.gov; it was assigned NCT00223080 as the registration number. Written informed consent and counseling was conducted as described previously [1], [2] and the protocol was reviewed by the ethics committees of the Thai Ministry of Public Health, the Royal Thai Army, Mahidol University, and the Human Subjects Research Review Board of the U.S. Army Medical Research and Materiel Command. It was also independently reviewed and endorsed by the World Health Organization, the Joint United Nations Program on HIV/AIDS, and by the AIDS Vaccine Research Working Group of the National Institute of Allergy and Infectious Diseases at the US National Institutes of Health. The manufacturers were full trial collaborators and were a part of the Phase III trial steering committee.

### Specimens

The initial pilot studies were performed using various sets of plasma from the RV144 participants which were selected randomly, evenly balanced for men and women, and derived from participants at visit 1 (pre-bleed), visit 8 (week 26 after the first immunization [two weeks after the last immunization]), and visit 9 (52 weeks). The pilot studies were performed with plasma sets C (SZP, PB), A and L (GT, BFH), and Z (MR, NK). Subsequently, case-control plasma specimens, described above, were tested for the primary and secondary variables selected as described above and in [2].

### Assays

#### ELISA for cyclic peptides and recombinant gp120 (NK)

**Figure 1:**
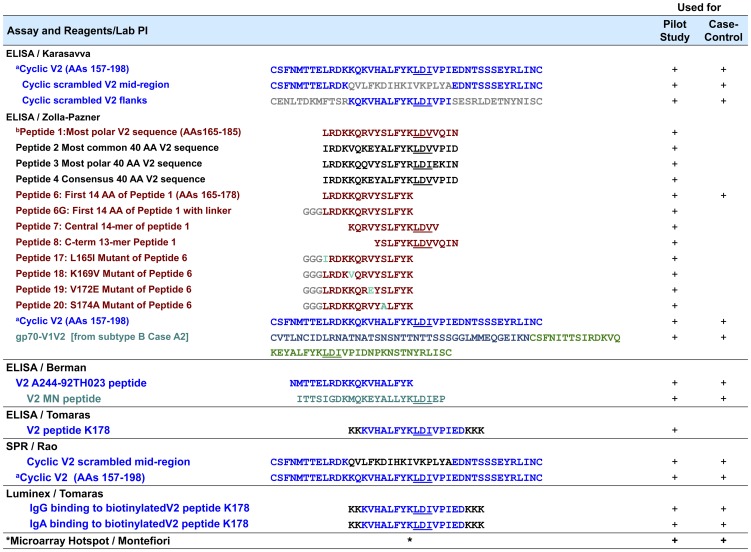
V2-related Reagents and Assays Used in the Pilot and Case-Control Analyses. The superscripts denote the following: ^a^ “Cyclic V2 (AAs 157–198)” was used in assays in three labs as shown. Throughout this table, blue V2 sequences are identical to the subtype E 92TH023 used in the prime; gray represent scrambled sequences or linkers; brown represents the sequence the central AAs in an extremely polar V2 in subtype A strain QB585.2102M.Ev1v5.C (http://www.hiv.lanl.gov) with individual mutations shown in cyan; black represents sequences chosen for particular properties, as described; green represents the V1V2 from subtype B Case A2 [20] and the central 23-mer of V2 from subtype B strain MN. ^b^ All peptides were biotinylated at the N-terminus with the exception of peptide K178 and peptide V2 A244-92TH023 which were biotinylated at the C-terminus. * Multiple V2 peptides from various strains (see text).

This assay was previously described [2]. Briefly, U-bottom ELISA plates were coated with either 1 µg/ml of cyclic peptide (**Figure 1**) or with 3 µg/ml of AIDSVAX recombinant gp120 immunogen A244 or MN. After washing, two-fold serial dilutions of plasma at an initial dilution of 1∶100 or, alternatively, anti-V2 human mAbs 2158 or 697-D [18], [19] were used at concentrations of 0.002–10 µg/ml. Color was developed with HRP-conjugated goat anti-human IgG and substrate, and read at A405 nm. The background value was determined from wells that did not contain recombinant proteins or peptides.

#### Biotinylated linear peptide ELISAs (SZP)

This assay was previously described [2]. Briefly, StreptaWell ELISA plates (Roche) were coated with 1 µg/ml of one of several N-terminus biotinylated linear V2 peptides (**Figure 1**); the plates were washed and incubated with RV144 plasma specimens diluted 1∶100 in RPMI medium containing 15% fetal bovine sera. Alkaline phosphatase (AP)-conjugated goat anti-human IgG and diethanolamine substrate were used to develop color which was read at A405 nm. At each step, every well contained 50 µl; at least two experiments were performed with each peptide/specimen combination.

#### Binding ELISA with gp70-V1V2 (SZP)

This method was previously described [2]. Briefly, plates were coated with 1 µg/ml gp70-V1V2 (**Figure 1** and [20]), washed, and then incubated for 1.5 h at 37°C with RV144 plasma diluted 1∶100 in RPMI containing 15% fetal bovine sera. After further washing, bound Abs was visualized using AP-conjugated goat anti-human IgG and diethanolamine substrate, and read at 405 nm. At each step, every well contained 50 µl; at least two experiments were performed.

V2 *Linear Peptide ELISA (PB)*. These assays were performed as previously described [21]. Briefly, plates were coated with 0.5 µg/well of peptide (**Figure 1**) and incubated overnight at 4°C. Three-fold dilutions of test sera were run in duplicate using a starting dilution of 1∶30. HRP-labeled anti-human IgG and substrate (OPD) were used to develop color.

#### ELISAs of linear and cyclic peptides and gp70-V1V2 (BFH)

Direct binding ELISAs were conducted as previously described [2] in 384-well ELISA plates coated with 2 ug/ml of linear or cyclic V2 peptides or gp70-V1V2 and incubated with three-fold serial dilutions of plasma at a starting dilution of 1∶50, followed by washing and incubation with 10 µl of HRP-conjugated goat anti-human Ig secondary Ab and substrate (SureBlue Reserve). Plates were read at 450 nm.

#### Overlapping peptide “hotspot” microarray assay (DM)

The arrays measured reactivity with 15-mer linear peptides with 12 residue overlaps. Raw peptide microarray data were processed and analyzed as described in [2]. Peptide sequences were provided by LANL to cover the entire gp160 HIV Env from six HIV-1 Group M subtypes (A, B, C, D, CRF01_AE and CRF02_AG) for a total of 1423 peptides. The specific peptides were determined by LANL's method for generating the mosaic peptide set [22] and were manufactured by JPT Peptide Technologies (Berlin, Germany).

#### Surface plasmon resonance (MR)

Measurements were conducted with a Biacore T100 as previously described [2]. Briefly, lysozyme (reference surface) and streptavidin (for peptide capture) were immobilized onto CM5 chips. Biotinylated V2 peptides (1 µM) (**Figure 1**) were manually injected over the streptavidin-coated chip surface. Heat-inactivated plasma samples diluted 1∶50 were injected over the chip surface followed by a dissociation period, after which a 50 nM solution of affinity-purified γ-chain-specific sheep anti-human IgG was passed over the peptide coated-Ig bound surface. Non-specific binding was subtracted and data analysis was performed using BIAevaluation 4.1 software. Case-control samples were run in triplicate.

#### IgG and IgA binding multiplex assays (GT)

These assays were performed as previously described [2], [23] using peptide K178 which represents a linear portion of V2 from immunogens A244 and 92TH023 (**Figure 1**). HIV-specific Ab isotypes were detected with goat anti-human IgA and mouse anti-human IgG.

#### Statistical analyses

Immune parameters measured two weeks after the last immunizing dose were assessed as correlates of subsequent infection risk using the previously described statistical analysis plan [2]. Briefly, for each immune parameter, logistic regression accounting for the sampling design was used to estimate the odds ratio (OR) of infection, controlling for gender and baseline behavioral risk. The OR was estimated both for each immune parameter as a categorical variable, and, for variables with greater than 50% of vaccinees exhibiting a Positive response, as a quantitative variable (scaled to have a SD = 1). For the categorical analysis, if the Positive response rate is less than 50%, then the OR compares Positive vs. Negative responders. If the Positive response rate is 50–85%, then the OR compares High vs. Negative, where High is above the median of the positive responders. For Positive response rates >85%, the OR compares High vs. Low, where High and Low are the upper and bottom tertiles of the response for vaccine recipients. The statistical analysis plan was finalized before data analysis and is described in detail in Haynes et al [2].

The lasso model selection procedure [24] as implemented with the glmnet R package (http://www.r-project.org) was used to assess the ability of 12 V2 variables from **Table 1** to predict infection when included in a multivariate logistic model adjusting for gender and behavioral risk score. The cyclic V2 scrambled mid-region variable was excluded since it had no positive responses. Two of the variables with low response rates, IgA V2 A244 K178 peptide and V2 MN peptide, were dichotomized as 1 for response and 0 for non-response, while the remaining ten variables were included on a quantitative scale. The best parsimonious model was chosen based on the average area under the receiver operating characteristic curve on test data derived from 1,000 10-fold cross-validation splits.

**Table 1:** Response rate and odds ratios (ORs) calculated from the case-control specimens tested with 13 V2 variables.

				Quantitative		Categorical	
Assay	Investigator	Institution	Response Rate	OR^1^^,^ 2	P value	OR^1^^,^ 3	P value
**V2 cyclic peptides - ELISA**	Nicos Karasavvas	AFRIMS					
Cyclic V2 (AAs 157–198)			0.47	NA	NA	0.82	0.63
Cyclic V2 scrambled mid-region			0.00	NA	NA	NA	NA
Cyclic V2 scrambled flanks			0.91	0.76	0.10	0.84	0.66
**V2 cyclic peptides – SPR**	Mangala Rao	USMHRP					
Cyclic V2 (AAs 157–198)			NA	0.81	0.24	0.84	0.66
Cyclic V2 scrambled mid-region			NA	0.79	0.18	0.90	0.80
**V2 reagents – ELISA**	Susan Zolla-Pazner	New York University					
Cyclic V2 (AAs 157–198)			0.95	0.82	0.26	0.65	0.31
Biotin V2 Peptide 6 (AAs 165–178)			0.76	0.95	0.80	0.85	0.76
gp70-V1V2			0.64	0.70	0.06	0.43	0.06
**V2 linear peptides - ELISA**	Philip Berman	University of California, Santa Cruz					
V2 MN peptide			0.10	NA	NA	0.41	0.25
V2 A244-92TH023 peptide			0.95	0.90	0.57	0.88	0.74
**IgA and IgG Abs vs. V2 peptide – Luminex**	Georgia Tomaras	Duke University					
IgA V2 A244 K178 peptide			0.06	NA	NA	0.79	0.77
IgG V2 A244 K178 peptide			0.93	NA	NA	1.02	0.95
**Peptide Microarray**	David Montefiori	Duke University					
V2 Hotspot analysis			NA	0.64	0.03	0.32	0.02

1 Estimated odds ratios are computed using a logistic regression model accounting for the sampling design and adjusting for gender and behavioral risk score, as described in Haynes et al [2].

2 Estimated odds ratio per one standard deviation increment in the immune biomarker; not available (NA) if response rates, when applicable, are less than 50%. For example, the OR of 0.70 (ELISA binding to gp70-V1V2) means that for every higher SD value, the rate of infection is reduced by 30%, while the OR of 0.43 means that vaccinees with responses in the upper third had an infection rate 57% lower than vaccinees with responses in the lower third.

3 Estimated odds ratios comparing subgroups defined by high vs. low responses except for two (IgA V2 A244 K178 and V2 MN) which compare positive vs. negative response and one (biotin V2 peptide 6) which compares high vs. negative; not available (NA) for Cyclic V2 scrambled mid-region (ELISA) which has no positive responses.

## Results

### Antigenicity of the boosting immunogens used

In RV144, the V2 sequence in the recombinant ALVAC priming immunogen derived from subtype E strain 92TH023 was:

^157^ **CSFNMTTELRDKKQKVHALFYKLDIVPIEDNTSS.SEYRLINC** ^198^.

The V2 sequence in the protein boosting gp120 immunogen AIDSVAX E (strain A244) was:

**^157^CSFNMTTELRDKKQKVHALFYKLDIVPIEDNNDS.SEYRLINC^198^.**

The V2 sequence of the protein boosting gp120 immunogen AIDVAX B (strain MN) was:

**^157^CSFNITTSIGDKMQKEYALLYKLDIEPI.DN.DSTS.YRLISC^198^.**

Insertion of periods in the sequences allows for alignment. Numbering shown and used throughout this report is that assigned to strain HxB2 [25].

**Figure 2:**
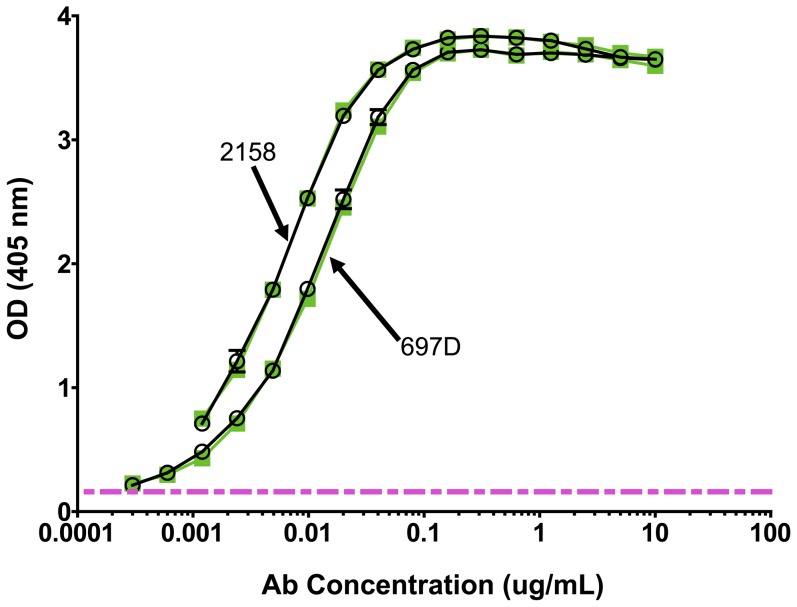
ELISA reactivity of human anti-V2 mAbs 697-D and 2158 with AIDSVAX subtype E (A244, green square) and AIDSVAX subtype B (MN, O) gp120 immunogens. The pink dashed line represents twice background levels.

The antigenic reactivity of the V2 region in AIDSVAX B and E was assessed using human anti-V2 mAbs 697D and 2158 [18], [19]. As shown in **Figure 2**, the titration curves for each of these mAbs with the two boosting immunogens could be superimposed, with half-maximal binding achieved at 0.0057 and 0.0055 µg/ml of mAb 697D, and 0.0041 and 0.0039 µg/ml of mAb 2158 vs. AIDSVAX A244 and AIDSVAX MN, respectively. This analysis suggests that, with respect to the highly conformational V2 epitopes recognized by these mAbs [7], [19], [26], the antigenicity of the A244 and MN gp120 immunogens are similar. Notably, these two mAbs also bind to gp70-V1V2 [7], [27].

### The V2 Ab response in RV144 can be detected with linear and cyclic peptides and V1V2-scaffolded antigens

**Figure 3:**
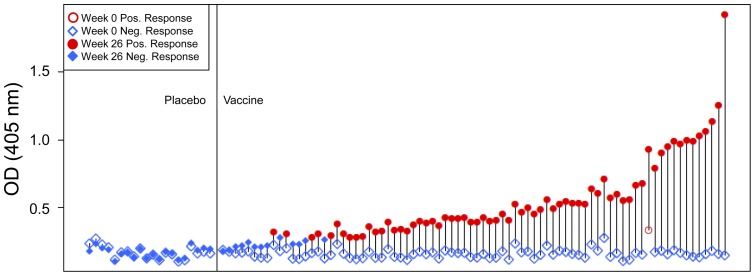
ELISA reactivity with gp70-V1V2 of plasma specimens used in the pilot studies of the RV144 clinical vaccine trial (Set C). The results from one of three experiments are shown. The open and filled blue diamonds depict negative responses at weeks 0 and 26, respectively. The open and closed red circles depict positive responses at weeks 0 and 26, respectively. Each vertical line connects a single patient's specimen drawn at Week 0 and Week 26. The specimens are ordered by the difference in reactivity between the Week 0 and Week 26 specimens, with the biggest increasers on the right. Plasma were tested at a final dilution of 1∶100, and a positive response was defined as being >0.283, the cut-off OD value which was defined as the mean +3 standard deviations based on values derived from vaccinees at week 0 (the pre-immunization time point).

**Figure 4:**
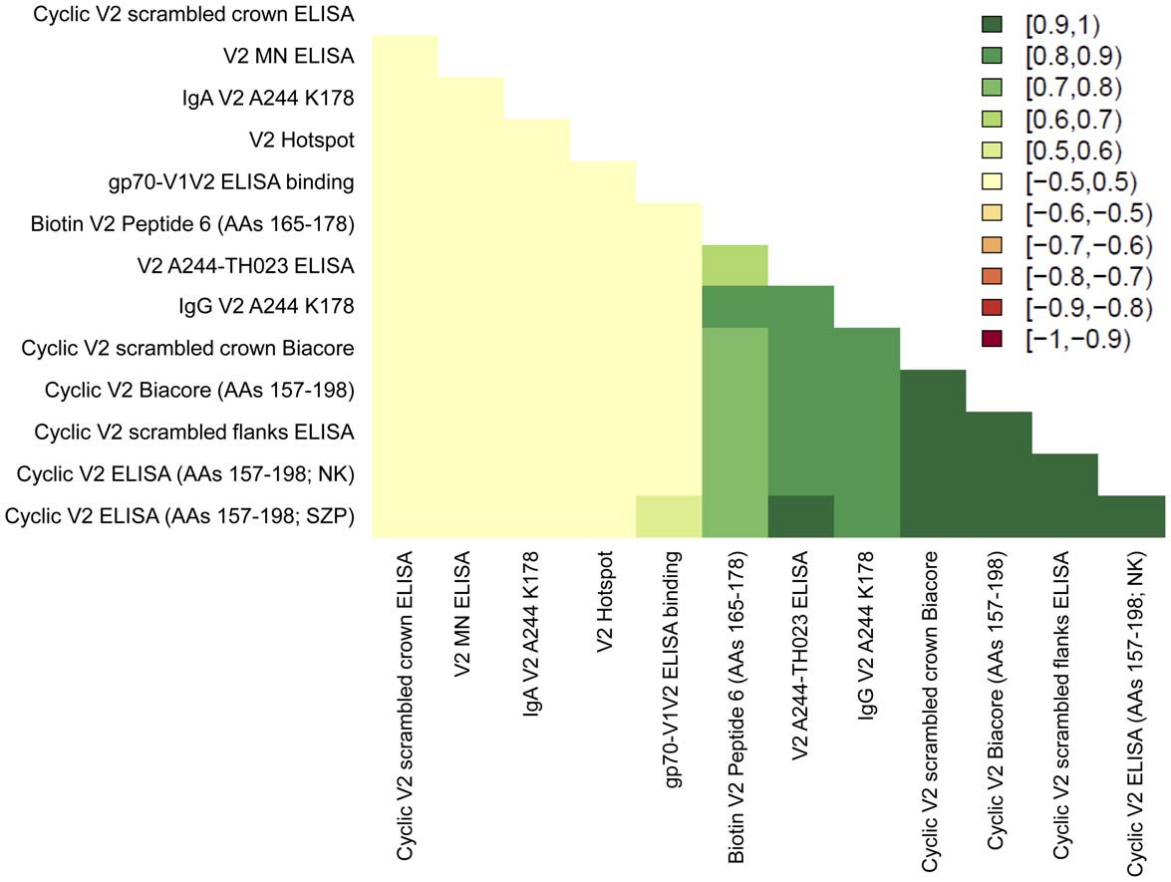
Spearman rank correlations between the V2 assays run in the case-control study.

A gp70-V1V2 scaffolded protein carrying the V1 and V2 variable regions from a subtype B strain, case A2, was previously described [20]. When Set C plasma specimens (from 20 placebo and 80 vaccine recipients) were tested in the pilot studies at a dilution of 1∶100, none of the specimens from the placebo recipients contained detectable Abs to gp70-V1V2. In contrast, the plasma of 67 of 80 (84%) vaccine recipients contained Abs reactive with this reagent (**Figure 3**). Moreover, the dynamic range of the assay was large, covering an optical density range from the cut-off (0.283 OD units, the mean +3 SD, based on values derived from vaccinees and placebo recipients at week 0, the pre-immunization time point) to 1.918. A relatively poor correlation was found between this assay and other assays that measured various V2 variables, suggesting that the reactivity measured with gp70-V1V2 may represent a unique Ab response (**Figure 4**).

The frequency of V2 responses detected with pilot study specimens derived from vaccinees varied with the assay used, ranging from 6% for IgA Abs reactive with a linear V2 peptide (K178) when measured by Luminex (see **Figure 1** and [2]) to 97% for IgG Abs reactive with an 92TH023 (subtype E) cyclic V2 peptide when measured by ELISA (see **Figure 1** and [27]).

### Delineation of the linear V2 epitopes recognized by plasma Abs from vaccines

**Figure 5:**
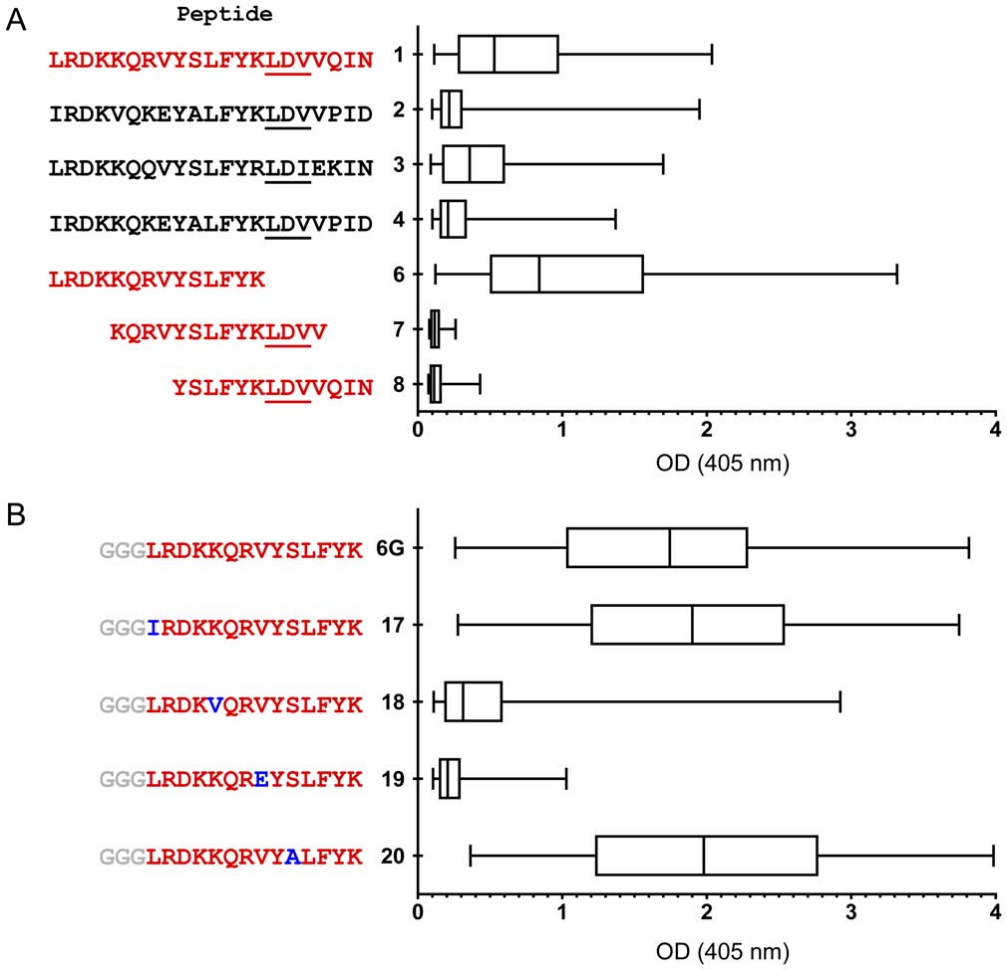
Boxplots showing ELISA reactivity of V2 peptides with plasma (diluted 1∶100) from 80 vaccinees' specimens from the pilot study (Set C). The distributions of the reactivities are shown where the left edge of each box equals the 25th percentile; the vertical line in each box is the 50th percentile, and the right edge of each box equals the 75th percentile. The boxplots were prepared using Prism, with “whiskers” showing the minimum and maximum responses. Panel A: Four 21-mer N-terminal biotinylated peptides (Peptides 1–4) were selected on the basis of a bioinformatics analysis of V2 sequences from the LANL HIV Database (see text). Panel B: A second peptide panel designed upon inspection of the AAs in Peptides 1–4 in Panel A revealed AAs that distinguish Peptides 1 and 3 from 2 and 4; these appear at positions 165, 169, 172, and 174. To maximally enhance the availability of the epitopes on the peptides used in the fine mapping of the V2 Abs, a spacer of three glycines was inserted between the biotin tag at the N-terminus of the peptide and the V2 sequences.

For fine mapping of linear V2 epitopes recognized by Abs in the plasma of RV144 vaccinees, four 21-mer peptides (Peptides 1–4 in **Figure 1** and **Figure 5A**) were selected on the basis of a bioinformatics analysis of V2 sequences from the LANL HIV Database. Peptide 1 was derived from the V2 of a strain with the highest number of polar AAs (subtype A strain QB585.2102M.Ev1v5.C from Kenya); this V2 was 38 AAs in length. Since V2 is most frequently 40 AAs in length [3], further analyses identified sequences from viruses containing V2 regions 40 AAs long: Peptide 2 represents the central 21 AAs of V2 in the most common naturally occurring sequence (derived from subtype B strain 878v3_2475). Peptide 3 is the V2 with the highest number of polar amino acids (from subtype A strain 01TZA341). Peptide 4 is the consensus V2 sequence among all viruses with V2 regions of 40 AAs.

**Figure 6:**
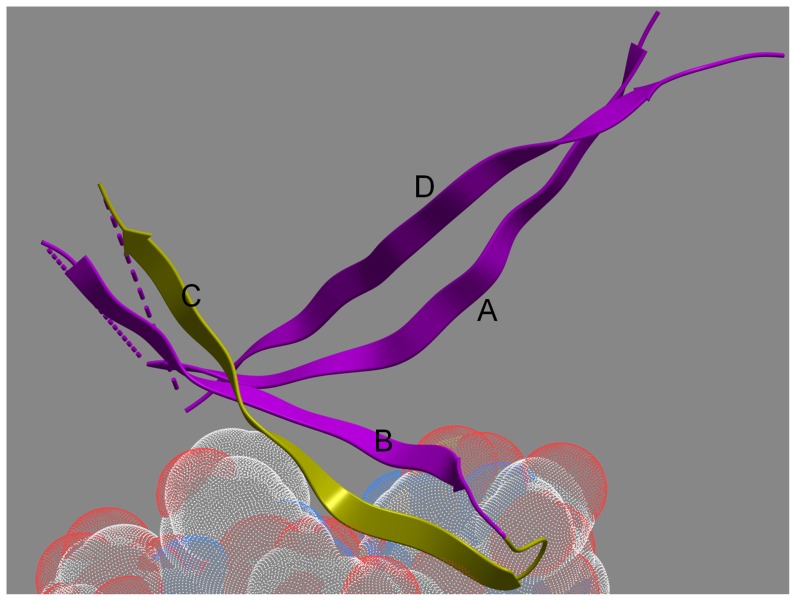
Ribbon diagram of the backbone fold of the V1V2 domain (purple and yellow ribbons) bound to the CDR H3 region of mAb PG9 (transparent stippled spheres are the atoms of the PG9 CDR H3, blue = nitrogen, red = oxygen, white = carbon). The ribbon backbone of AAs 165–176, which are identical to Peptide 6 (in **Figure 5**), is colored yellow. The strands are labeled A–D according to the convention established recently [28].

As illustrated by the ELISA data (**Figure 5A**), Peptide 1 was the most reactive with plasma from the RV144 vaccinees: 59% of plasma showed positive reactivity, i.e., above the cut-off based on the mean +3 SD of control plasma from placebo and vaccine recipients at Visit 1 (week 0). Peptide 3 was also reactive (68% positive), while Peptides 2 and 4 were much less reactive (10% and 23% positive, respectively). Three overlapping peptides (Peptides 6–8) were synthesized based on the sequence of the most strongly reactive Peptide 1. Results with the overlapping peptides showed that the epitope maps to the 14 residues in Peptide 6 containing AAs 165–178 (**Figure 5A**). The residues in this V2 region form the outer C strand of the ß-sheet folded domain of V1V2 (**Figure 6**) [28].

To distinguish the AAs that play a critical role in determining anti-V2 Ab reactivity, a further series of peptides was designed. Results with these peptides indicated that L165I (Peptide 17) and S174A (Peptide 20) replacements had little effect on reactivity (**Figure 5B**). In contrast, the K169V (Peptide 18) and the V172E (Peptide 19) replacements profoundly reduced the reactivity of the plasma, indicating that these two residues are critical for binding of V2 peptides by the vaccine-induced Abs. These data acquire enhanced importance in light of results showing that (a) sieve analysis showed that a residue other than K at position 169 was more frequent in breakthrough viruses in vaccine recipients vs. placebo recipients [29], and (b) glutamic acid (E) strongly predominates in subtype B whereas valine (V) predominates at position 172 in subtype E (unpublished data). Notably, the poor reactivity with Peptides 18 and 19 suggests that the V2 Ab response to this linear epitope was induced by the subtype E (A244) gp120 boosting immunogen rather than the subtype B (MN) immunogen.

### Comparison of V2 assays run in the case-control study

Based on the pilot studies, six assay types were chosen for measuring 13 variables with case-control specimens (**Table 1**). One of these (ELISA binding to gp70-V1V2) was chosen as a primary variable. The secondary variables provided by the 12 additional V2 assays were run in exploratory analyses with case-control specimens. The primary and secondary variables were chosen to represent assays whose results did not correlate with one another. The heat map in **Figure 4** represents the Spearman rank correlations between the V2 assays, and demonstrates that the primary variable, binding of IgG Abs to gp70-V1V2, correlated only weakly with ELISA binding to cyclic V2 (AAs 157–198) (Spearman correlation: 0.5–0.6). Analysis of the microarray “hotspot” data was not performed until after completion of the analysis of the initial pilot and case-control studies. Interestingly, the V2 hotspot variable correlates poorly with all of the other V2 variables.

**Figure 7:**
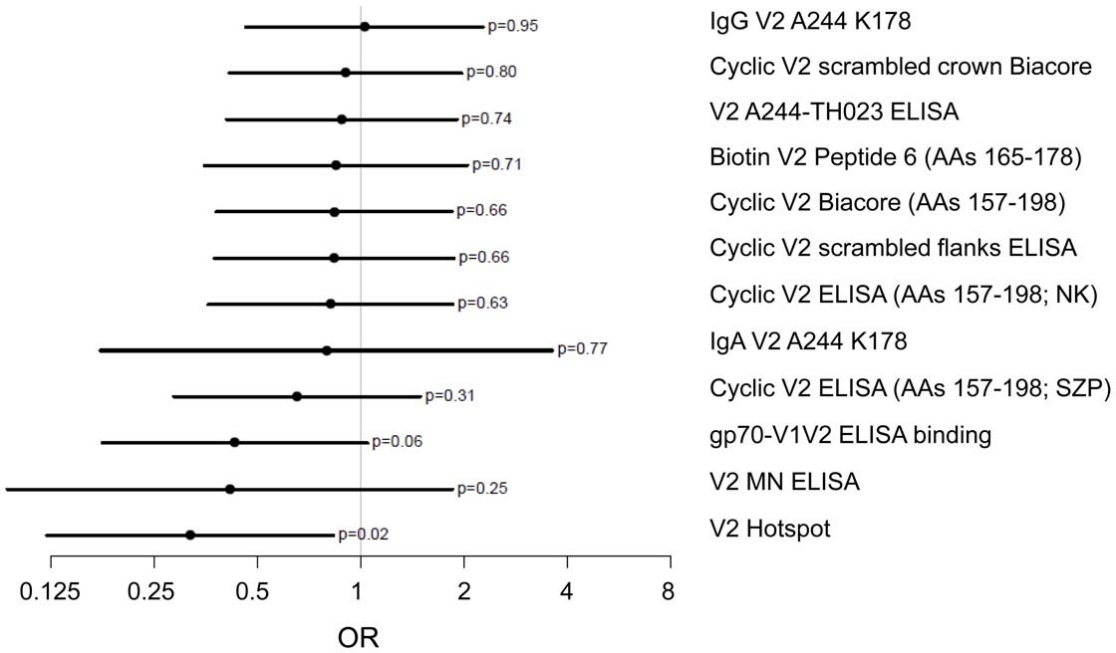
Estimated odds ratios (OR) and 95% confidence intervals for each of the V2 assays. Data are derived from the categorical analyses shown in **Table 1**. Estimated ORs compare subgroups defined by High vs. Low responses except for comparisons for analyses of IgA V2 A244 K178 and V2 MN which compare Positive vs. Negative responses. For evaluation of biotinylated V2 Peptide 6, comparison is between High and Negative responses.

**Figure 8:**
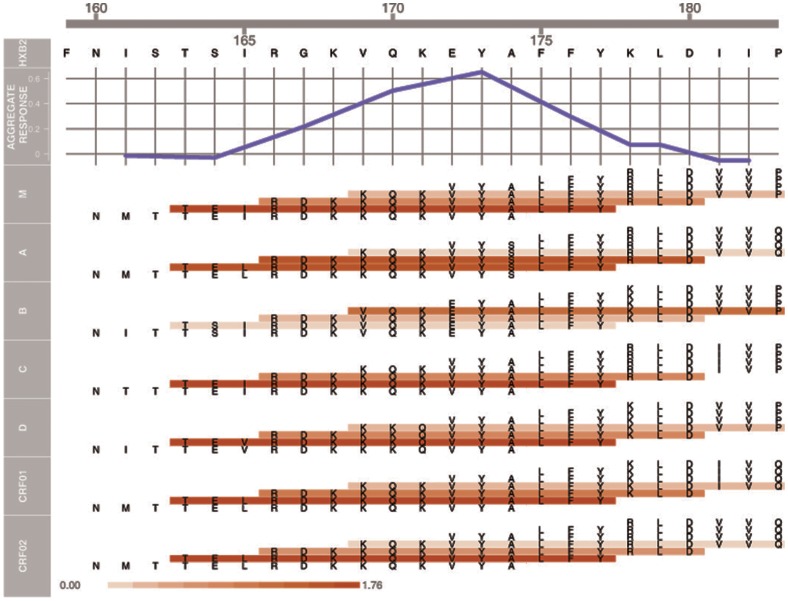
Hotspot analysis of the microarray data of V2 Abs in the plasma from vaccinees in the case-control study. The upper panel shows an aggregate response across all sub-types averaged across vaccinees as a function of the V2 sequence in subtype B strain HxB2. An individual aggregate response is computed using a sliding window mean statistic of 9 AAs, i.e., peptides with HxB2 positions of 9 contiguous AAs averaged together. In the lower portion of the figure, the actual sequence of each of the overlapping 15-mers spanning V2 positions 160–183 is shown. The seven sets of peptides represent the consensus V2 regions of HIV-1 Group M and of subtypes A, B, C, D, CRF01_AE and CRF02_AG. Peptides are colored according to their average response across all vaccinees, with a scale of 0 (white) to 1.76 (red). All responses are calculated using normalized intensities and by subtracting the intensities of baseline pre-bleeds.

For the univariate analysis, as in the multivariate analysis [2], statistical significance was approached or achieved with the primary variable of ELISA binding to gp70-V1V2 (p = 0.06 and p = 0.02, respectively). With one of the secondary variables (V2 microarray hotspot), significance was achieved (p = 0.03 quantitative, and p = 0.02 categorical, **Table 1**). The ORs calculated for each V2 variable are shown in **Figure 7** and **Table 1**. The univariate ORs for 11 of 12 V2 variables were ≤1, compatible with the hypothesis that V2 Abs played a role in reducing infection. When binding Abs were assessed with peptide microarrays using linear overlapping peptides covering the entire V2 region of seven major genetic subtypes, the lowest and most statistically significant OR was achieved (**Figure 7** and **Table 1**). The results of this hotspot analysis of the microarray data are shown in **Figure 8** for V2 residues 160–183. Most of the remaining C-terminal portion of V2 is poorly immunogenic [3], and similarly, there was little or no reactivity with peptides in the V1 region (unpublished data). The aggregate response (upper panel), shows that the V2 response is centered around residue Y^173^. The peptides with the strongest reactivity encompass residues 163–177 (lower panel), which matches the results from the independent ELISA data described above. It is also noteworthy that the weakest reactivity was detected with the subtype B subset of V2 peptides, confirming the poor reactivity with Peptides 18 and 19 (**Figure 5B**) which contain canonical subtype B V2 substitutions at positions 169 and 172. Interestingly, the V2 peptide representing the sequence of vaccine strain MN (subtype B) also contains residues at positions 169 and 172 that are not present in the A244 (subtype E) boost. Only 24 of 246 vaccinees' specimens reacted with the V2 MN peptide, again confirming the poor reactivity with the subtype B linear V2 peptide, however, notably, only two of these 24 vaccinees were infected, resulting in an OR for Positive vs. Negative responders of 0.41 (**Table 1** and **Figure 7**). The 0.25 p-value reflects the low power of these data due to the very few positive responders and could also be due to poor sensitivity of the assay since the results were reported as endpoint titers after a starting dilution of 1∶30.

## Discussion

In this study, we have probed the results achieved with the entire panel of V2 assays used in the RV144 pilot and case-control studies in order to understand more fully the nature of the V2 Ab response and why the high response to epitopes in this region is associated with a lower rate of infection in vaccinees. The data presented include *all* of the data available from both the pilot and the case-control specimens that describe the V2 Ab response induced by the vaccine.

Our studies document at least two types of V2 Abs induced by the RV144 vaccine: Abs reactive with a scaffolded V1V2 protein, gp70-V1V2, and Abs specific for linear and cyclic V2 peptides. Studies with human mAbs suggest that these may be non-overlapping Ab populations since human mAbs such as 697D and 2158 react with conformational V1V2 epitope(s) carried by glycosylated gp70-V1V2 containing a subtype B sequence, but not with linear or cyclic (non-glycosylated) peptides [7], [18], while mAb CH59 reacts with linear V2 peptides but not with gp70-V1V2 (unpublished data). The primary variable that correlated with reduced risk of infection measured Ab activity in ELISA with gp70-V1V2. This reagent retains a conformation presented *in vivo* during infection since it is recognized by Abs in sera of infected individuals and was used for the selection of two mAb-producing hybridomas from the cells of HIV-infected individuals which are broadly cross-reactive with diverse envelopes and which neutralize several Tier 1 pseudoviruses [7], [19].

The reactivity of vaccinees' Abs with overlapping V2 peptides also correlated with reduced risk of infection (**Table 1**), generating the hypothesis that Abs to linear V2 epitopes were also involved in reducing the rate of HIV infection. The data generated with various linear V2 peptides indicate that: (a) the dominant immunogenic linear V2 epitope in the RV144 vaccine encompasses residues 165 to 178; (b) the V2 Abs were induced primarily by the subtype E A244 rather than the subtype B MN gp120 boost; (c) the V2 Abs were cross-reactive with V2 peptides derived from several subtypes, (d) the dominant linear V2 epitope was located in the C β-strand of the V1V2 complex (**Figure 6** and [28]), (e) residues K^169^ and V^172^, were critical for the binding of vaccinees' plasma Abs to V2 peptides, and (f) the V2 epitope includes K^169^ which was identified by sieve analysis to be mismatched in vaccine breakthrough infections [29].

It is noteworthy that the single primary variable showing an inverse correlate of infection risk in the RV144 case-control study was Ab reactivity with gp70-V1V2 which contains the V1V2 sequence of case A2, a subtype B strain [20] which carries both the V169 and the E172 residues that reduce reactivity of vaccinees' Abs with V2 peptides (**Figure 5B**). Notably, however, as shown above, vaccinees' plasma do react with subtype B-derived linear V2 peptides, though with less potent and less frequent reactivity than with V2 peptides from other subtypes. The data with gp70-V1V2 and the linear peptides are not incompatible and may suggest that the effective Ab populations are those which are broadly cross-reactive, targeting conformational and linear epitopes shared by diverse HIV-1 subtypes. Indeed, these data, together with bioinformatics data on V2 [3] and studies of the preferential gene usage of VH families by V2-specific mAbs [7], support the presence of conserved and immunologically cross-reactive elements in the V2 region. The role of shared structures and antigenic determinants in the variable regions of the envelope in inducing potentially protective Ab responses is also suggested by the involvement of the V2 and V3 regions as components of the epitopes recognized by the class of potently neutralizing Abs that target quaternary epitopes and proteoglycans on the envelope spike [30]–[37].

The explanation for the strong induction of V2 Abs by the A244 subtype E gp120 immunogen compared to the weak response induced by the MN subtype B gp120 despite the similar antigenicity of the two proteins may be due to the fact that the V2 domain of A244 rgp120 is more immunogenic than that of MN rgp120, and that it usually takes three immunizations with protein, given at appropriate intervals, to elicit good seroconversion to the V2 domain [38]. In addition, the vaccinees were primed with the vCP1521 vector which carries the *env* gene of CRF01_AE, whose V2 region is nearly identical to that of the V2 of the A244 gp120 protein boost, and is extremely divergent from the V2 carried by the MN gp120 boost. Thus, it would appear that the Ab response to the CRF01_AE V2 was primed more effectively than the response to the V2 of subtype B. To address these issues, further studies of V2 responses with specimens from other vaccine trials, e.g., VAX003 and VAX004, are underway, along with assays using additional peptides, proteoglycans, and epitope-scaffolded proteins.

Finally, the mechanisms by which anti-V2 Abs may reduce HIV infection have yet to be understood. As noted, anti-V2 mAbs can neutralize many Tier 1 pseudoviruses in the TZM.bl assay [7]. It is possible that they mediate broader neutralizing activity than is detected in this particular assay. Plasma samples from RV144 neutralized some Tier 1 viruses in the TZM.bl assay and in a more sensitive assay with A3R5 cells, however no neutralization of Tier 2 viruses was detected in either assay [39]. Since V2 can be detected on the surface of virions [40] and infected cells [41], these Abs may also mediate various other anti-viral functions such as ADCC, ADCVI, virolysis, virus opsonization, virus aggregation, etc. Along with current studies of the potential biologic functions of V2 Abs, assessment is on-going to test several hypotheses, including those that postulate that anti-V2 Abs prevent conformational changes in the envelope necessary for binding to CCR5, and that these Abs may, or may not, prevent binding of the envelope to α4β7 [13], [42]–[44]. Interestingly, after vaccination of non-human primates with Ad26 and MVA containing SIVsm543 inserts, results from a low dose intra-rectal heterologous SIVmac251 challenge identified a potential V2 correlate of protection [45]. While there are many differences between these experimental results and those emanating from the RV144 clinical vaccine trial, the analogous Ab responses in both associated with reduced infection may provide a means to illuminate the postulated mechanism(s) by which V2 Abs reduce the risk of infection.
